# Aortic Dissection and Hypotension Without Cardiac Tamponade: A Case Report

**DOI:** 10.7759/cureus.44418

**Published:** 2023-08-30

**Authors:** Ryuichi Ohta, Chiaki Sano

**Affiliations:** 1 Community Care, Unnan City Hospital, Unnan, JPN; 2 Community Medicine Management, Shimane University Faculty of Medicine, Izumo, JPN

**Keywords:** japan, general medicine, rural healthcare, sex disparities, comprehensive assessment, cerebral hypoperfusion, vertigo, atypical presentation, hypotension, ascending aortic dissection

## Abstract

Ascending aortic dissection is typically characterized by severe chest or back pain. However, its presentation can be atypical, leading to diagnostic challenges, especially in settings where classic symptomatology may not be evident. In this report, we described the case of a 74-year-old woman who presented to the emergency room of a rural community hospital with chief complaints of vertigo, nausea, and vomiting, without the classic symptoms of chest or back pain associated with aortic dissection. Despite initial treatment for autonomic dysregulation, the patient’s symptoms persisted. Subsequent comprehensive assessments, including computed tomography angiography, revealed an ascending aortic dissection extending to the bilateral common carotid arteries. This atypical presentation, characterized by cerebral hypoperfusion and systemic hypotension without tachycardia, emphasizes the need to maintain a high suspicion index, even in the absence of hallmark symptoms. This case underscores the importance of considering the possibility of ascending aortic dissection in patients with nontraditional symptoms. Recognizing these atypical presentations is crucial for timely intervention, especially in rural settings with limited advanced diagnostic tools. This case also highlights potential sex disparities in symptom presentation, emphasizing the need for clinicians to recognize nontraditional symptoms in women. Rapid identification, evaluation, and management are imperative to prevent severe outcomes, and a multidisciplinary approach has proven to be the most effective in such cases.

## Introduction

Aortic dissection is a serious condition characterized by a tear in the aortic wall. This tear allows blood to flow between the arterial layers, compromising perfusion to multiple organs [1]. Aortic dissection can precipitate a life-threatening condition known as cardiac tamponade, in which blood or fluid fills the space between the heart muscle and its outer sac, leading to hypotension and shock, as well as aortic dissection-induced hypotension in the absence of cardiac tamponade [2]. Internal bleeding from the tear can cause a marked decrease in blood pressure. If the dissection extends to the coronary arteries, it may lead to myocardial ischemia or infarction, thereby impairing the pumping efficiency of the heart [3]. Additionally, severe pain from aortic dissection can trigger a vagal response, further reducing heart rate and blood pressure.

Interestingly, hypotension is a frequent manifestation of aortic dissection, but it may be considered as somewhat atypical, as aortic dissection is more commonly linked to hypertension. Nevertheless, in acute cases, hypotension can indicate imminent rupture, significant blood loss, or involvement of vital organs, making it a critically emergent condition [4]. Managing aortic dissection with hypotension but without cardiac tamponade necessitates patient stabilization, blood pressure control, and definitive treatment such as surgical repair and endovascular intervention [3].

Diagnosing aortic dissection in cases with hypotension but without chest and back pain or pericardial effusion is challenging. In this case report, we described and presented a case of an older woman who primarily presented with vertigo, nausea, and vomiting. Although the patient initially manifested hypotension without the classic signs of aortic dissection, comprehensive assessments eventually confirmed the diagnosis [2]. This report also explores the differential diagnosis of hypotension in the absence of tachycardia and the complexities of diagnosing aortic dissection without the hallmark symptoms.

## Case presentation

A 74-year-old woman presented to the emergency department of a rural community hospital complaining of vertigo, nausea, and vomiting on the day of her visit. She reported experiencing transient vertigo and headache seven days prior, after which she consulted her primary care physician and was prescribed 500 mg acetaminophen every 6 hours for pain relief. She denied experiencing headache; chest, back, or abdominal pain; dyspnea; palpitations; syncope; or diarrhea and had no recent history of trauma, overseas travel, or contact with individuals infected with any virus or bacteria. Her medical history was significant for hypertension, and she was taking a daily dose of amlodipine (5 mg).

During the physical examination, the vital signs were as follows: blood pressure, 53/24 mmHg; pulse rate, 67 beats/min; body temperature, 35.8 °C; respiratory rate, 16 breaths/min; and oxygen saturation, 96% on room air. The patient was alert and oriented to time, place, and person but exhibited mild drowsiness. Notable findings included profuse cold sweats in the extremities and a collapsed jugular vein. Apart from an altered level of consciousness, no other neurological deficits were observed. Examination of the chest, abdomen, and skin revealed no abnormalities. Laboratory investigations did not indicate elevated cardiac markers such as creatinine kinase and troponin I; inflammatory markers, such as C-reactive protein; and white blood cell counts (Table 1).

**Table 1:** Initial laboratory data of the patient eGFR: estimated glomerular filtration rate; CK: creatine kinase; CRP: C-reactive protein; TSH: thyroid-stimulating hormone

Parameter	Level	Reference
White blood cells	7.40	3.5-9.1 × 10^3^/μL
Neutrophils	72.1	44.0-72.0%
Lymphocytes	18.7	18.0-59.0%
Monocytes	6.4	0.0-12.0%
Eosinophils	2.1	0.0-10.0%
Basophils	0.7	0.0-3.0%
Red blood cells	3.45	3.76-5.50 × 10^6^/μL
Hemoglobin	10.0	11.3-15.2 g/dL
Hematocrit	30.3	33.4-44.9%
Mean corpuscular volume	87.6	79.0-100.0 fL
Platelets	17.9	13.0-36.9 × 10^4^/μL
Total protein	6.4	6.5-8.3 g/dL
Albumin	4.0	3.8-5.3 g/dL
Total bilirubin	0.8	0.2-1.2 mg/dL
Aspartate aminotransferase	21	8-38 IU/L
Alanine aminotransferase	10	4-43 IU/L
Alkaline phosphatase	115	106-322 U/L
γ-Glutamyl transpeptidase	15	<48 IU/L
Blood urea nitrogen	23.3	8-20 mg/dL
Creatinine	0.66	0.40-1.10 mg/dL
eGFR	66.0	>60.0 mL/min/L
Serum Na	140	135-150 mEq/L
Serum K	3.5	3.5-5.3 mEq/L
Serum Cl	108	98-110 mEq/L
CK	26	56-244 U/L
Troponin I	0.0021	0.000-0.029 ng/mL
TSH	1.16	0.35-4.94 μIU/mL
Free T4	1.26	0.70-1.48 ng/dL
CRP	0.03	<0.30 mg/dL
Urine test		
Leukocyte	Negative	Negative
Nitrite	Negative	Negative
Protein	Negative	Negative
Glucose	Negative	Negative
Urobilinogen	Normal	
Bilirubin	Negative	Negative
Ketone	Negative	Negative
Blood	Negative	Negative
pH	7.0	
Specific gravity	1.011	

**Figure 1:**
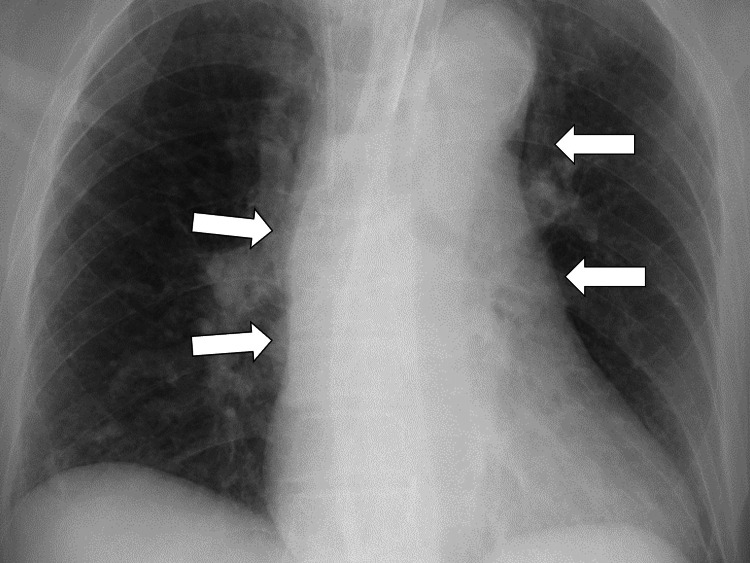
Chest radiography not revealing any mediastinal widening, pleural cap, and no displaced calcium sign (white arrows).

The chest radiograph did not reveal any mediastinal widening (Figure 1).

**Figure 2:**
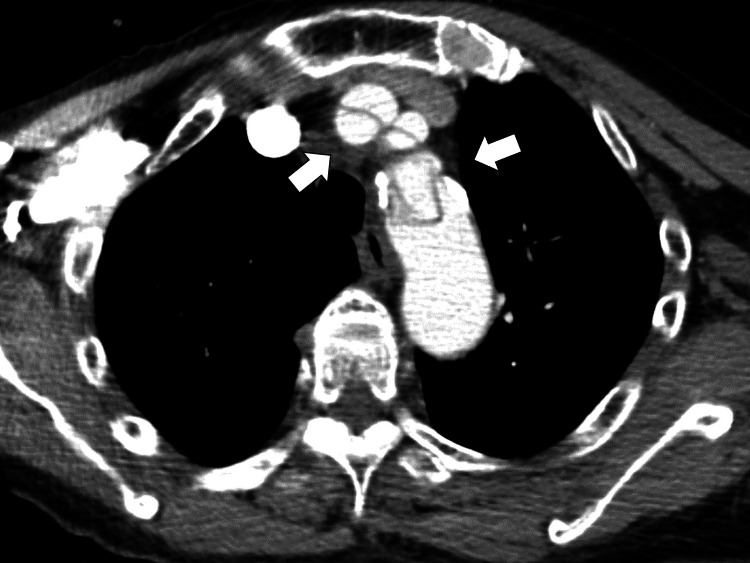
Computed tomography angiography of the chest revealing an ascending aortic dissection extending to the bilateral common carotid arteries (white arrows).

Initially, the patient was diagnosed with autonomic dysregulation. Fluid resuscitation was initiated, leading to a transient improvement in blood pressure. However, the patient continued to experience lightheadedness, and the blood pressure decreased. A potential cardiac etiology was suspected; electrocardiography and echocardiography were performed. Neither investigation revealed significant cardiac dysfunction or pericardial effusion. Considering the persistent cold sweats, hypotension without tachycardia, and other unexplained symptoms, the differential diagnosis was expanded to include brainstem hypoperfusion causing sympathetic hyperexcitability. One potential etiology that could encompass both clinical scenarios is aortic dissection. Chest computed tomography (CT) angiography revealed ascending aortic dissection extending to the bilateral common carotid arteries (Figure 2).

The patient was diagnosed with ascending aortic dissection based on the CT findings. The presenting symptoms of vertigo and hypotension were attributed to reduced perfusion to the brainstem and systemic circulation. The patient was then transferred to a tertiary care facility for surgical intervention. Postoperatively, the patient had symptom resolution.

## Discussion

This case underscores the need to consider the diagnosis of ascending aortic dissection in patients presenting with hypotension without tachycardia, even when classic symptoms, such as chest or back pain, are lacking. We investigated the essential differential diagnoses of hypotension without tachycardia and emphasized the importance of identifying aortic dissections without relying solely on typical manifestations.

Cerebral hypoperfusion due to ascending aortic dissection can manifest as hypotension without tachycardia. As the dissection extends proximally, it can jeopardize the origin of vital vessels branching from the aorta to nourish the brain, including the brachiocephalic trunk and left carotid artery [5]. The involvement of these vessels can compromise cerebral blood flow. Considering the brain’s sensitivity to blood supply alterations, compromised brainstem perfusion may induce hyposympathetic states, culminating in hypotension [6]. The urgency of accurate diagnosis and immediate management cannot be overemphasized, as it decreases the risk of irreversible neurological deficits [7]. Ascending aortic dissections can precipitate cerebral hypoperfusion through myriad mechanisms, each with grave implications for patient prognosis. Therefore, immediate medical evaluation is paramount in these cases.

Regarding ascending aortic dissection, atypical symptoms require heightened clinical vigilance, especially in rural settings where advanced diagnostic resources may be unavailable. Although the paradigmatic presentation of this condition often encompasses acute, intense chest or back pain, frequently described as a “tearing” or “ripping” sensation, many patients deviate from this typical pattern [8]. Some patients may only experience unconventional symptoms, potentially stalling a definitive diagnosis and intervention. Understanding these unusual presentations is indisputable for timely therapeutic measures. The female patient presented with dizziness and hypotension without pain in the present case. Women do not always experience pain during cardiovascular events [9,10]. Beyond sex disparities, the unique pathophysiology, in this case, offers insights into such an atypical presentation.

This case illustrates that general physicians should be aware of ascending aortic dissection, even without red flags of chest or back pain. Conventional medical pedagogy and diagnostic schemas for aortic dissections may inadvertently overlook patients with atypical signs, potentially leading to protracted diagnoses and suboptimal outcomes [5,6]. This may be especially true for clinicians in resource-limited settings, emphasizing the necessity for exhaustive clinical assessments that consider both traditional and nontraditional presentations [11]. Additionally, given the potential sex-based differences in symptomatology, clinicians need to remain alert to unconventional symptom presentations in women [12]. An integrative, multidisciplinary approach utilizing cardiology, radiology, and emergency medicine expertise is crucial for the rapid identification and management of such severe conditions. This holistic strategy will bolster patient safety, minimize complications, and be lifesaving by facilitating prompt therapeutic measures. As systems-specific specialists, general physicians should be educated and keen on various unexplained symptoms and logically consider the pathophysiology of patients' conditions, not missing critical diseases such as this case [13,14].

## Conclusions

Ascending aortic dissection can present with a myriad of symptoms, some of which may not align with the conventional understanding of the condition. Our case of a 74-year-old woman presenting with vertigo, nausea, and hypotension but devoid of hallmark chest or back pain symptoms emphasizes the importance of maintaining a holistic clinical approach. Clinicians must rely on comprehensive evaluations, recognizing both typical and atypical manifestations, especially in settings where high-end diagnostic tools may be limited. Potential sex-based differences in symptom presentation may further complicate the diagnosis. This report underscores the need for timely diagnosis and intervention in atypical cases of ascending aortic dissection and a multidisciplinary approach to enhance patient outcomes.
